# Rise of the specialized onco-ribosomes

**DOI:** 10.18632/oncotarget.26231

**Published:** 2018-10-16

**Authors:** Kim R. Kampen, Sergey O. Sulima, Kim De Keersmaecker

**Affiliations:** Kim De Keersmaecker: Department of Oncology, KU Leuven - Catholic University of Leuven, LKI - Leuven Cancer Institute, Leuven, Belgium

**Keywords:** onco-ribosome, IRES, BCL-2, RPL10-R98S, ribosomopathy

Ribosomes are essential cellular nano-machines tasked with the synthesis of all cytoplasmic proteins. Historically, the structure of ribosomes — four ribosomal RNA (rRNA) molecules and 81 ribosomal proteins (RPs) assembled into a 4.3 mDa particle — has been assumed to be invariable. By extension, identical ribosomes and the associated translational machinery were believed to passively promote equal translation of all mRNAs that are present in the cell. However, this static, monolithic view on the ribosome is now being gradually adapted towards a more malleable and varied ribosomal reality. Ribosomal diversity was recently supported by the observation that the translational apparatus can differ in RP composition and in the identity of ribosome-associated proteins in mouse embryonic stem cells. Another layer of ribosomal diversity is provided by differences in RP and rRNA modifications. Notably, such structurally unique ribosomes possess specialized functions, as they display selective mRNA translation [1]. These paradigm-shifting discoveries have raised numerous exciting and important questions. For example, do specialized ribosomes hold the key to understanding diseases associated with defective ribosomes, such as the ribosomopathies and the numerous cancers with RP gene mutations which have recently been described [2]? If so, can the unique functions of such onco-ribosomes be therapeutically targeted? To this end, our recent work on the R98S hotspot mutation in ribosomal protein L10 (RPL10-R98S), recurrently found in pediatric T-cell leukemia (T-ALL), may provide some answers [3].

**Figure 1:**
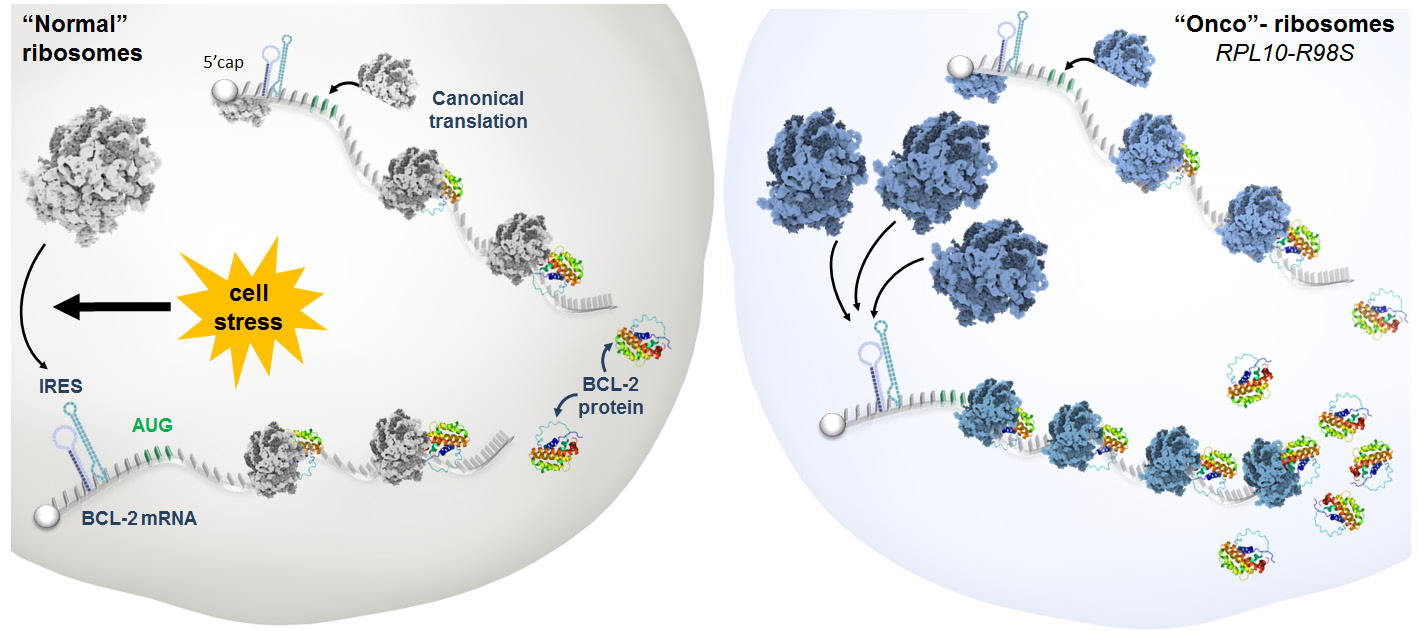
The specialized function of an onco-ribosome leading to the overexpression of BCL-2

We noticed that leukemia cells carrying the RPL10-R98S mutation showed increased cell fitness, endowing ribosome-mutant cells with a selective survival advantage. This phenotype was associated with overexpression of the anti-apoptotic protein B-cell lymphoma 2 (BCL-2). Intriguingly, BCL-2 mRNA levels were unaffected in RPL10-R98S cells, suggesting a post-translational mechanism. Indeed, we found that RPL10-R98S ribosomes showed stronger association with the BCL-2 mRNA as compared to wild type ribosomes. Translation of BCL-2 is known to be controlled by an Internal Ribosomal Entry Site (IRES) under cellular stress [4]. IRES elements are mRNA secondary structures located upstream of the open reading frame which can recruit ribosomes and direct them to initiate protein synthesis independent of the canonical 5’ cap-driven translation. These elements were first discovered in viruses, where they are used to lure ribosomes onto viral messages and to overcome the rate-limiting step of 5’ cap-dependent translation initiation. More recently, such IRES-driven translation has also been described in eukaryotes, where it is favored under conditions of cellular stress, when cap-dependent translation is compromised. We discovered that RPL10-R98S cells display a specific preference for translating BCL-2 messages due to the BCL-2 IRES acting akin to a magnet for RPL10-R98S ribosomes, a remarkable example of ribosome specialization (Figure 1). Moreover, the increased capability of RPL10-R98S ribosomes to constitutively drive IRES-dependent BCL-2 translation was not dependent on cellular stress. This specialized function of the RPL10-R98S onco-ribosome is characterized by a functional cellular advantage, resulting in a dependence of RPL10-R98S positive T-ALL cell models and patient samples on the survival protein BCL-2. This is turn renders such samples sensitive to the clinically available BCL-2 inhibitor venetoclax, providing an example of how the specialized function of an onco-ribosome can be therapeutically exploited [3].

Additional studies support that different types of specialized ribosomes display differential IRES-mediated translation. For example, modifications of the rRNA methylation pattern as a consequence of TP53 inactivation increases the translation of IRES-containing cellular mRNAs in cancer cells [5]. Conversely, DKC1 mutations affecting another type of rRNA modification — pseudouridylation — prevents the translation of IRES-containing mRNAs, including the tumor suppressor genes *TP53* and *CDKN1B* and the anti-apoptotic factors *BCL2L1* and *XIAP* [6-8]. The DKC1 gene is also mutated in the ribosomopathy Dyskeratosis Congenita, a cancer predisposition syndrome. These studies indicate that the combination of specialized onco-ribosomes and IRES elements can modulate translation of a proteome subset in cancer cells. IRES elements are structurally diverse, explaining why different onco-ribosomes have a preference for particular IRES elements. In agreement with this, translation of other IRES containing messages besides BCL-2 was not significantly altered in our RPL10 R98S cell models.

In addition to IRES elements, a number of other cis-acting mRNA elements have been described, including: RNA G-quadruplex structures, 5′ terminal oligopyrimidine tract motifs, translation inhibitor element, pyrimidine-rich translational element, cytosine-enriched regulator of translation, and programmed ribosomal frameshifting (PRF) signals. Many of these are enriched on mRNAs encoding cancer associated genes. For example, PRF signals are enriched in genes of the JAK-STAT signaling cascade, which is associated with overexpression of JAK-STAT proteins and a sensitivity to JAK-STAT inhibitors in RPL10-R98S cells [9]. It is tempting to hypothesize that the regulatory potential of many of these mRNA elements is influenced by the interactions with different types of ribosomes — an intriguing avenue for future studies.

While the RPL10-R98S mutation is highly specific to pediatric T-ALL, mutations in other ribosomal proteins (i.e. RPL5) are more broadly found in a variety of different cancers [10]. It will be interesting to see whether RPL5-mutated onco-ribosomes also display a specialized function across different cancers, or whether their function is disease-specific. As it is becoming apparent that a diversity of ribosomes exists, it is likely that additional specialized onco-ribosomes will be discovered in the near future. Identifying these can be a daunting task however, as the current methodologies to study protein translation are limited. Understanding the structure and function of oncogenic ribosomes, along with discovering their inhibitors, represent the next ambitious and important challenges in the ribosome and cancer fields.
